# Evaluation of the Impact of Orthodontic Treatment on Patients' Self-Esteem: A Systematic Review

**DOI:** 10.7759/cureus.48064

**Published:** 2023-10-31

**Authors:** Rashad I. Shaadouh, Mohammad Y. Hajeer, Ahmad S. Burhan, Mowaffak A. Ajaj, Samer T. Jaber, Ahmad Salim Zakaria, Khaldoun M.A. Darwich, Ossama Aljabban, Youssef Latifeh

**Affiliations:** 1 Department of Orthodontics, Faculty of Dentistry, University of Damascus, Damascus, SYR; 2 Department of Orthodontics, Faculty of Dentistry, Al-Watanyia Private University, Hama, SYR; 3 Department of Orthodontics, School of Dental Sciences, Universiti Sains Malaysia, Kelantan, MYS; 4 Department of Oral and Maxillofacial Surgery, Faculty of Dentistry, University of Damascus, Damascus, SYR; 5 Department of Endodontics and Restorative Dentistry, Faculty of Dentistry, University of Damascus, Damascus, SYR; 6 Department of Internal Medicine, Faculty of Medicine, University of Damascus, Damascus, SYR

**Keywords:** fixed orthodontic treatment, orthodontic treatment, self-concept, adults, adolescent, fixed treatment, orthodontic, psychological, self-esteem

## Abstract

Malocclusion may affect interpersonal relationships, self-esteem (SE), and psychological well-being, weakening patients' psychological and social activities. Several studies investigated the effect of orthodontic treatment on these social and psychological aspects, such as SE. However, the direct relationship between SE and orthodontic treatment has not yet been confirmed. This systematic review aimed to evaluate the existing evidence in the literature concerning the influences of orthodontic treatment on patients’ SE systematically and critically. An electronic search in the following databases was done in September 2022: PubMed®, Web of Science™, Scopus®, Embase®, GoogleTM Scholar, Cochrane Library databases, Trip, and OpenGrey. Then, the reference list of each candidate study was checked for any potentially linked papers that the electronic search might not have turned up. Inclusion criteria were set according to the population/intervention/comparison/outcome/study design (PICOS) framework. For the data collection and analysis, two reviewers extracted data separately. The risk of bias 2 (RoB-2) and the risk of bias in non-randomized studies (ROBINS-I) tools were used to assess the risk of bias for randomized controlled trials (RCTs) and non-RCTs, respectively. The grading of recommendations assessment, development and evaluation (GRADE) approach was employed to evaluate the quality of the evidence for each finding. Sixteen studies (five RCTs, seven cohorts, and four cross-sectional) were included in this review. Unfortunately, the results could not be pooled into a meta-analysis. Only six studies have reported an increase in SE after orthodontic treatment (P<0.05 in these studies). No agreement between the included studies was observed regarding the influence of fixed orthodontic treatment, gender, or age on SE. The quality of evidence supporting these findings ranged from very low to low. There is low evidence indicating that fixed orthodontic treatment can improve patients' SE. In addition, unclear data are available about the influence of patients' gender and age on SE after orthodontic treatment. Therefore, high-quality RCTs are required to develop stronger evidence about this issue.

## Introduction and background

Malocclusion is a common public health problem, which causes physical and psychological implications for patients and influences their daily life [1]. Many studies have shown its negative impact on social perceptions [2]. It may affect appearance, interpersonal relationships, self-esteem, and psychological health, weakening patients' psychological and social activities, such as smiling, emotion, and social contact [3,4].

On the other hand, the orthodontic treatment itself and its appliance may affect the psychological and social activities of patients due to the appearance of these devices [5,6], their effect on speech [7-9], the accompanying pain and discomfort [10-12], and the associated functional impairment [11].

Due to the growing appreciation of the impact of dentofacial problems on social and psychological health [13], orthodontists have argued that the aesthetically pleasing appearance of teeth and associated soft tissue leads to greater self-esteem (SE) and social health [14,15]. As a result, several studies investigated the social and psychological aspects of malocclusion and orthodontic treatment, such as oral health-related quality of life (OHRQoL) [1,5] and SE [13,16,17] to understand the impact of malocclusion on patients’ lives and to develop effective orthodontic care that improves patient’s attitudes toward treatment and their self-concept and SE [18].

Generally, the self-concept embodies the answer to the question, "Who am I?" [19]. Piers determined self-concept as a set of attitudes people have about themselves that describe and evaluate their behavior [20]. Moreover, self-concept was defined by Beane et al. as the perceptions that a person has of oneself in relation to individual attributes and the various roles performed by the person [21]. Self-concept cannot be described as positive or negative since it is irrelevant to value judgments and represents only a description of the perceived self. In contrast, SE refers to the estimation that a person makes about the description of one's self-concept and, more precisely, to what extent one is satisfied or dissatisfied with his/her self-concept, in whole or in part. Thus, King argued that SE and self-concept represent two discrete dimensions [22].

Self-esteem was defined as a multifaceted notion, for which Harter developed a tool to measure both global and specific self-worth [23]. Explicit SE refers to beliefs and values in particular domains, such as school competence or close friendship, whereas global SE refers to one's perception and assessment of oneself as a person [24]. It has been stated that adolescents with little SE have a higher chance of developing worse mental and physical health, poorer economic well-being, and higher levels of criminal behavior in adulthood [25].

Although this well-known and accepted correlation between SE and malocclusion, the direct relationship between SE and orthodontic treatment has not been confirmed yet; while several studies show that orthodontic treatment may improve SE scores at the end of treatment [13,17,26], others have found no differences in SE after the completion of orthodontic treatment [24,27,28]. Thus, there is no clear evidence about the effect of orthodontic treatment on self-esteem. Additionally, no previous systematic review was performed on this topic. Therefore, this systematic review aimed to evaluate the existing evidence in the literature concerning the influences of orthodontic treatment on patients' SE systematically and critically. The focused review question was "How does orthodontic treatment affect patients’ self-esteem?"

## Review

Materials and methods

Scoping Search

A scoping search was conducted in the PubMed database before designing the final systematic review protocol to verify the existence of any systematic reviews with comparable objectives and to investigate potentially relevant papers. No literature reviews regarding how orthodontic treatment affects patients' SE were found as a result of this search. Several articles that were related to the topic of this review were found.

Eligibility Criteria

The participants/interventions/comparisons/outcomes/study design (PICOS) framework was used to define the inclusion criteria.

Participants: Healthy patients of all ages and malocclusions, both males and females, of all racial groups undergoing orthodontic treatment were included.

Interventions: Any orthodontic treatment using fixed or removable orthodontic appliances.

Comparisons: In the case of two- or three-arm comparable studies, the comparison group may be any group of patients who did not undergo any form of orthodontic treatment or a group of patients being treated with another orthodontic technique different from that in the interventional group or a group of subjects with normal occlusion.

Outcomes: Patients’ SE after orthodontic treatment is measured by the Rosenberg scale, Harter’s self-perception profile, the global negative self-evaluation, or any other validated scale for SE assessment. The effect of the type of orthodontic treatment and patients’ age and gender on SE is determined.

Study design: In English, randomized controlled trials (RCTs) or non-RTC (CCTs), prospective cohort studies, and cross-sectional studies were included without time of publication restrictions.

Sources and Search Strategy

PubMed®, Web of Science™, Scopus®, Embase®, Google TM Scholar, Cochrane Library, PsychINFO, Trip, and OpenGrey databases were electronically searched in September 2022 without time limits. The details of the electronic search strategy for each database are presented in Appendix 1. The keywords used in the search strategy are listed in Appendix 2. The reference list of each candidate study was checked for any potentially linked papers that the electronic search might not have turned up.

Study Selection

After electronically removing the duplicated papers retrieved from the databases and manual searches using the Endnote™ reference management software program (Clarivate Analytics, Philadephia, PA, USA), the titles and abstracts of articles were assessed. Two reviewers (RIS and MYH) independently evaluated the suitability of each article in light of the selection criteria. Then, the entire text of all articles that potentially meet the inclusion criteria was assessed by the same two reviewers or could not reach a clear judgment based on the title or summary. Articles were excluded if they failed to satisfy one or more qualifying criteria. In case of disagreement and a conversation did not result in agreement, a third reviewer (ASB) was consulted.

Data Collection Process

The following data were among the information extracted from the included articles in this review and organized into summary tables: author's name, year of publication, country, study design, comparison, sample size (male/female), mean age, malocclusion, type of orthodontic treatment, questionnaire employed, questionnaire administration time, main finding, and p-value.

Risk of Bias Assessment of the Studies

First, the risk of bias of each included study was assessed by the two reviewers (RIS and MYH) separately using Cochrane's risk of bias tool for randomized trials (RoB2) [29] and ROBINS-I tool for non-RCTs [30]. Second, the judgments of both reviewers were compared. In case of disagreement, and a conversation did not result in agreement, a third reviewer (MAA) was consulted to help reach a decision. For RCTs, the five domains of the RoB2 tool were judged as having a high, low, or unclear risk of bias.

After that, the overall risk of bias for each study was determined according to the following criteria: a low risk of bias if all fields were assessed as having a low risk of bias; a moderate risk if one or more fields were evaluated as having an unclear risk of bias; and a high risk of bias, if one or more fields were assessed as being at high risk of bias.

For the non-RCTs, the seven domains of the ROBINS-I tool were rated as having a low, moderate, critical, no information, or serious risk of bias. After that, the overall risk of bias for each study was determined according to the following criteria: low risk of bias if all fields were assessed as having a low risk of bias; a moderate risk if all fields were assessed as having a low or moderate risk of bias; serious risk of bias if one or more fields were assessed as having a serious risk of bias, but no critical risk of bias in any field; critical risk of bias if one or more fields were assessed as having a critical risk of bias; and no information when there was a lack of information in one or more key bias categories and no overt indication that the study is at serious or critical risk of bias.

The Quality of the Evidence

Based on the grading of recommendations assessment, development and evaluation (GRADE) approach, the strength of the evidence was rated as high, moderate, low, or very low for each outcome. The quality of the evidence of each outcome was assessed by the two reviewers (RIS and MYH) separately. After that, the judgments of both reviewers were compared. In case of disagreement and a conversation was not resolved, a third reviewer (MAA) was consulted to help reach a decision.

Synthesis of Results

Due to the qualitative nature of the data, meta-analysis was not feasible. Instead, a thematic synthesis approach was employed to synthesize the data. Thematic analysis is a suitable method for qualitative research [26]. The findings were summarized based on significant and prominent themes. Consequently, the following thematic headings were identified: (1) effect of orthodontic treatment on SE; (2) the effect of type of orthodontic treatment on SE; and (3) the effect of age and gender on SE.

Results

Literature Search Flow and the Retrieved Studies

**Figure 1:**
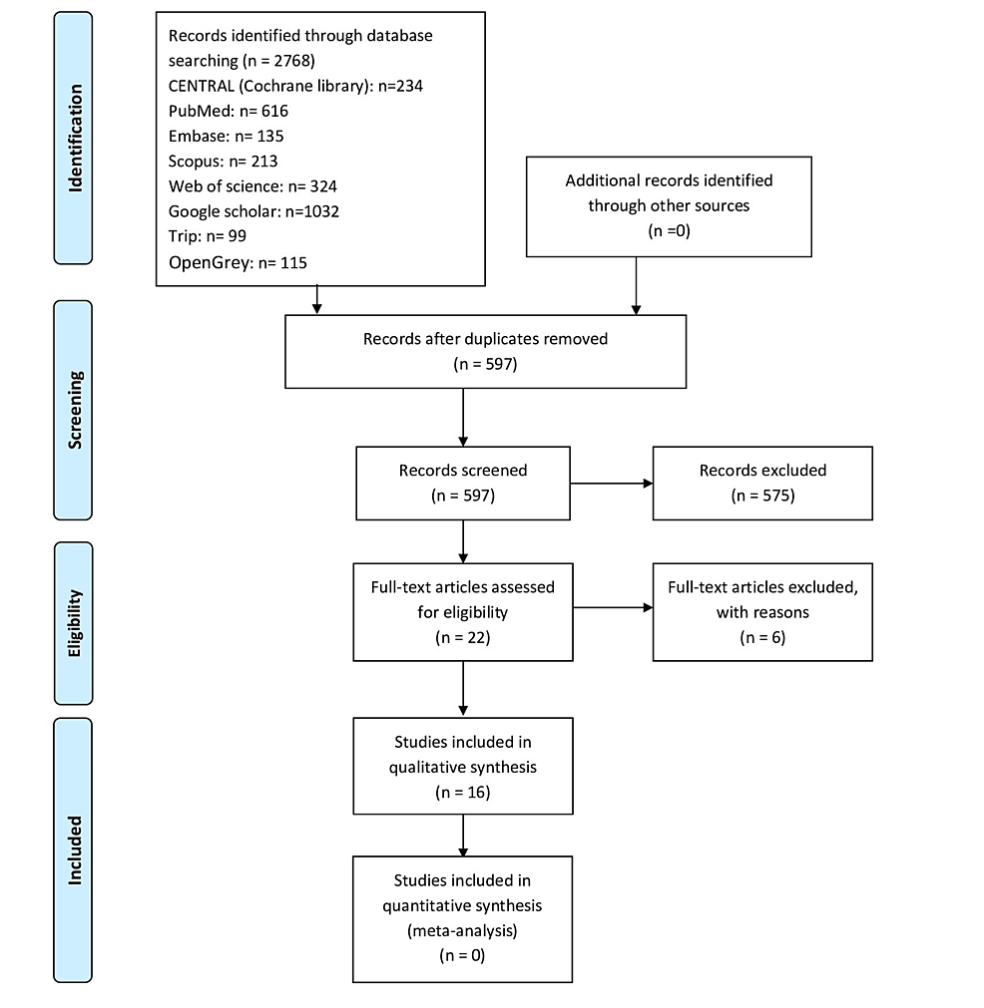
PRISMA 2009 flow diagram of the included studies

The electronic search in the databases and reference lists yielded 2,768 references. After removing duplicate references, 597 citations were carefully checked. A total of 575 documents were removed based on checking the titles and abstracts, and then the eligibility of 22 full-text records was evaluated. As a result, 16 studies were included in the systematic review [13,17,24,26-28,31-40], and six were excluded. The reasons for exclusion are given in Appendix 3. Figure 1 shows the PRISMA flow chart for the processes of selection and inclusion.

Studies’ Characteristics

Table 1 summarizes the characteristics of the included studies. Out of these trials, five were RCTs [26,27,37,38,40], seven studies were cohort studies [13,24,28,32,34,35,39], and the other four studies had a cross-sectional design [17,31,33,36]. All of them were in English. These studies were carried out across seven countries, including the UK [13,34,35,37,38,40], Brazil [26,33], Korea [17,28], the USA [27,31], Spain [36,39], Belgium [24], Norway [32].

**Table 1:** Characteristics of the included studies in the systematic review RCT: Randomized clinical trials, CSS: cross-sectional, TG: Treated group, CG: control group, FixApp: fixed orthodontic appliances, RemoApp: removable orthodontic appliances, FuncApp: functional appliances, FM: face mask, IOTN: Index of orthodontic treatment need, PAR: Peer assessment rating, IOCN: Index of complexity, outcome and need, NR: not reported

Study setting	Methods		Participants		Interventions				Results	
Author, Year, Country	Study design	Type of comparison	Patients (female/male) and age range (years)	Malocclusion	Type of orthodontic treatment	Malocclusion assessment	Used questionnaire	Questionnaire administration time	Main findings	P-valve
Pithon et al. 2021 [26], Brazil	RCT	Treated group vs Control group	44 adult patients (31 female/13 male), age: 17-49, TG: 22 patients, CG: 22 patients	Skeletal class I and Angle Class I or II malocclusions with missing lateral incisors	Fixed appliances	NR	Rosenberg’s Self-Esteem Scale (RSES)	TG: T1: before orthodontic treatment, T2: after orthodontic treatment, CG: T1: at baseline, T2: after 12 months	The spacing resulting from missing maxillary lateral incisors had a negative impact on the self-esteem of the participants, while orthodontically closing those spaces had a positive impact on this aspect	< 0.001
Avontroodt et al. 2019 [24], Belgium	Cohort	Pre-treatment vs Post-treatment	T0: 326 adolescents (172 girls/154 boys), age: 11-16 years, T2: 123 adolescents	NR	Fixed appliances	IOTN	Harter’s Self-Perception Profile for Adolescents (SPPA)	T0: baseline, T1: namely 1 year after the start of treatment (T1), T2:1 month after the end of treatment	There was no statistically significant difference in all questionnaire scores between T0 and T2 Global self-esteem acts as a stable construct during orthodontic treatment	0.0564
Choi et al. 2017 [28], Korea	Cohort	Pre-treatment vs Post-treatment	T0: 66 adult patients (36 female/30 male), age: 19-39 years, mean age: 24.2 ± 5.2 years, T2: 66 adult patients	Class I, II, or III with or without premolar extraction	Fixed appliances	IOTN	Rosenberg Self-esteem Scale (RSES)	T0: at baseline, T1: 12 months after treatment initiation, T2: debonding	There was no statistically significant difference in all questionnaire scores between T0 and T2	> 0.05
de Couto Nascimento et al. 2016 [33], Brazil	Prospective cross-sectional design	Pre-treatment vs Post-treatment	T1: 102 adult patients (77 female/25 male), age: 18-66 years, T2: 102 adult patients	Malocclusions caused by dental losses and agenesis	Fixed appliances	NR	Rosenberg’s Self-Esteem Scale(RSE)	T1: early orthodontic treatment (1–3 months of treatment), T2: after leveling and alignment phase (minimum of 8 months of treatment)	Orthodontic treatment causes a significant increase in patients’ self-esteem and QoL	< 0.001
Mandall et al. 2016 [40], UK	RCT	Treated group vs Control group	T1:73 patients (39 female/34 male), TG: 35 patients, CG: 38 patients (mean age: 9± 0.8 years), T2: 65 patients (33 female/32 male), TG: 33 patients (mean age: 15 years ± 10.3 months), CG: 32 patients (mean age: 15.3 years ± 10.1 months)	Class III malocclusion	FM	NR	Piers Harris questionnaire	T1: at baseline, T2: at 6-year follow-up	Early protraction facemask treatment does not seem to confer a clinically significant psychosocial benefit	0.48
Romero-Maroto et al. 2015 [36], Spain	Cross-sectional	Treated group vs Control group	170 adult patients (100 female/70 male), mean age: 29.80 ± 9.55 years, TG: 85 patients, CG: 85 patients	Class I, Class II, and Class III malocclusion with anterior malalignment and no need for extractions, dental crowding >6 mm	Fixed appliances	NR	Rosenberg’s Self-Esteem Scale (RSES)	T1: before treatment, T2: after 3-6 months of treatment	No significant differences were found in relation to self-esteem between the groups	0.839
Johal et al. 2014 [13], United Kingdom	Cohort	Pre-treatment vs Post-treatment	T0: 61 adult patients (48 female /13 male), age: 18-71 years, mean age: 41.2, T4: 60 adult patients	NR	Fixed appliances	IOTN	Rosenberg’s Self-Esteem Scale (RSE)	T0: baseline, T1: after 1 month, T2: after 3 months, T3: after 6 months, T4: post-treatment	Undergoing fixed orthodontic therapy appeared to have a significant improvement in self-esteem.	0.002
Seehra et al. 2013 [35], UK	Cohort	Pre-treatment vs Post-treatment	T0: 27 patients (14 female/13 male), mean age: 14.6 (±1.5) years, T1: 27 patients	Classes I, II, or III	FixApp/FuncApp/Retainers	IOTN	Harter’s Self-Perception Profile	T0: Pre-treatment, T1: post-treatment	There were no significant differences in the pre-and posttreatment scores of the participants on the Harter measure of self-esteem scale	NR
Mandall et al. 2012 [38], UK	RCT	Treated group vs Control group	T1:73 patients (39 female/34 male), TG: 35 patients (mean age: 8.7±0.9 years), CG: 38 patients (mean age: 9±0.8 years), T3: 63 patients (33 female/30 male), TG: 30 patients (mean age: 12.1±0.9 years), CG: 33 patients (mean age: 12.3±0.8 years)	Class III malocclusion	FM	NR	Piers Harris questionnaire	T1: at baseline, T2: at 15-month follow-up, T3: at 3-year follow-up	There were tiny changes in self-esteem over time and no statistically significant increase in self-esteem as a result of protraction facemask treatment	0.56
Jung et al. 2010 [17], Korea	Cross-sectional	DB: after debonding of fixed appliances) group FO: FOA treatment group RO: During or finished ROA treatment group NO: No orthodontic treatment group	4,509 patients (2,944 female/1,565 male), age: 12-15 years	Crowding/protrusion/other types of malocclusion	FixApp/RemoApp	NR	Rosenberg’s Self-Esteem Scale (RSE)	1 week before the clinical examinations	Anterior crowding causes low self-esteem in adolescent girls. FO or RO treatment could not improve self-esteem during treatment; however, after fixed treatment, significantly higher self-esteem was observed in the girls	NR
Mandall et al. 2010 [37], UK	RCT	Treated group vs Control group	T1:73 patients (39 female/34 male), TG: 35 patients (mean age: 8.7±0.9 years), CG: 38 patients (mean age: 9±0.8 years), T2: 69 patients, TG: 33 patients (mean age: 10±0.9 years), CG: 36 patients (mean age: 10.3±0.8 years)	Class III malocclusion	FM	NR	Piers Harris questionnaire	T1: at baseline, T2: at 15-month follow-up	There was no increased self-esteem (Piers–Harris score) for treated children compared with controls	0.22
Show et al. 2007 [34], UK	Cohort	Group 1: Prior need for treatment in 1981 –treatment received by 2001. Group 2: Prior need for treatment in 1981 – no treatment by 2001 Group 3: No prior need for treatment in 1981 – no treatment by 2001 Group 4: No prior need for treatment in 1981 – treatment received by 2001	T0: 1,018 patients, Age: 11-12 years, T1: 332 (188 female/144 male), age: 29.6-32.4 years	NR	NR	ICON	Rosenberg Self-Esteem Scale (RSE)	T0: at baseline (1981) T1: third follow up (2001)	there appears to be a significant effect of orthodontic treatment on self-esteem in later life. The group of participants who had a prior need in 1981, but who did not receive treatment, had lower self-esteem in 2001 than the control group (no prior need, no treatment) and significantly lower self-esteem than the prior need group who received treatment; this last group had the highest mean self-esteem in 2001	< 0.01
Birkeland et al. 2000 [32], Norway	Cohort	ROA group vs FOA group vs Control group	T1: 359 children, mean age: 11 years, T2: 224 children (120 girls/104 boys), mean age: 15 years RemoApp, G: 16 patients/FixApp, G: 51 patients, CG: 157 patients	NR	FixApp/RemoApp	IONT PAR	The Global Negative Self-Evaluation Scale (GSE)	T1: at baseline (age: 11 years old), T2: 15 years old	An overall improvement in GES score over the 4-year period was found. A gender difference was found	<0.001
Varela et al. 1995 [39], Spain	Cohort	Pre-treatment vs Post-treatment	T1: 40 adult patients (37 female/3 male), age: 18-42 years, T3: 40 adult patients (37 female/3 male)	moderate to severe malocclusions	Fixed appliances	Three independent orthodontists	Tennessee Self-Concept Scale (TSCS)	T1: before treatment, T2: after 6 months of treatment, T3: from 1 to 4 weeks after the end of active treatment.	Changes across the three measurement periods were not significant	NR
Albino et al. 1994 [27], USA	RCT	Treated group vs Control group	T1: 93 patients (46 female/47 male), age: 11-14, TG: 44, CG: 49, T5: 76 patients, TG: 39, CG: 37	Mild-to-moderate malocclusions	Fixed appliances	Treatment Priority Index	Coopersmith Self-esteem Inventory Rosenberg Self-image Inventory	T1: before treatment, T2: during treatment (8-10 months after began), T3: on termination of active treatment, T4: 6 months after termination, T5: 1 year after termination	treatment did not affect the subjects' self-esteem	NR
O'Regan et al. 1991 [31], USA	Cross-sectional	Pre-treatment group vs Post-treatment group vs Control group	220 patients (144 female/76 male), pre-TG: 97 patients, mean age: 13.3. post-TG: 45 patients, mean age: 15.8, CG: 78 patients, mean age: 13.1	NR	Fixed appliances	NR	The Piers and Harris self-rating questionnaire	NR	Improvement in dental and/or facial aesthetics does not necessarily lead to an increase in self-esteem	NR

From these 16 studies, 6,287 participants were included (3,996 females and 2,291 males). All these studies included a mixture of both genders, and there was no single-gender study. Six studies evaluated adult patients between 17 and 71 years [13,26,28,33,36,39]. Children and adolescents aged between 7 and 15 were evaluated in nine studies [17,24,27,31,32,35,37,38,40]. Noteworthy, the Show et al.'s cohort study followed patients over 20 years with age at baseline 11-12 years, and the mean age at the final follow-up assessment was 31.25 years [34].

To assess patients’ SE, the Rosenberg’s Self-Esteem (RSE) questionnaire was used in eight studies [13,17,26-28,33,34,36], and the Piers and Harris self-rating questionnaire in four studies [31,37,38,40]. In addition, Harter’s Self-Perception Profile for Adolescents (SPPA) was used in two studies [24,35]. Coopersmith Self-esteem Inventory, the Global Negative Self-Evaluation Scale (GSE), and Tennessee Self-Concept Scale (TSCS) were also used by Albino et al. [27], Birkeland et al. [32], and Varela et al. [39], respectively, to assess SE.

Self-esteem was studied with several types of malocclusions among the included studies; patients with mild-to-moderate malocclusion were assessed by Albino et al. [27]; moderate-to-severe malocclusion by Varela et al. [39]; Class III malocclusion in children patients was studied in three studies [37,38,40]; and cases with dental loss or agenesis and missing lateral incisors were evaluated by de Couto Nascimento et al. [33] and Pithon et al. [26], respectively. Jung's study focused on patients with crowding or protrusion or both of them [17]. Romero-Maroto et al.'s trial included patients with anterior crowding less than 6 mm with Class I, Class II, or Class III malocclusion and no need for extractions [36]. On the other hand, patients with Class I, II, or III with or without premolar extraction were included in Choi et al.'s and Seehra et al.'s studies [28,35]. In contrast, the other included studies lacked this information about malocclusion type [13,24,31,32,34,35].

The Index of Orthodontic Treatment Need (IOTN) scale defined the treatment need and assessed malocclusion in five studies [13,24,28,32,35]. Meanwhile, the Index of Complexity, Outcome and Need (ICON), and Treatment Priority Index were used in two studies by Show et al. and Albino et al., respectively [27,34]. Three independent orthodontists assessed malocclusion severity in Varela et al.'s trial [39].

Among the included studies, fixed orthodontic appliances were used in patients’ treatment in nine trials [13,24,26-28,31,33,36,39]; a face mask with a bonded maxillary acrylic expansion device was used in three trials [37,38,40]; and, in the other two trials, a mixture of fixed or removable orthodontic appliances was used in different groups [17,32]. In the trial reported by Seehra et al., 59% and 23% of patients were treated with class II functional appliances, followed by fixed appliances and fixed appliances only, respectively [35].

Out of the 16 included studies in this systematic review, eight compared treated patients vs the control group (untreated patients) [26,27,32,34,36-38,40]. However, in the other six single group before-after studies, patients' SE was compared between pre- and post-treatment [13,24,28,33,35,39]. O'Regan et al., in a cross-sectional study, compared patients’ SE between three groups (pre-treatment group vs post-treatment group vs control group) [31]. Lastly, the study of Jung divided patients into four groups (DB: after debonding of fixed appliances group, FO: fixed appliances treatment group, RO: During or finished removable appliances treatment group, NO: No orthodontic treatment group) and compared patients’ SE between them [17].

Risk of Bias in the Included Studies

**Figure 2:**
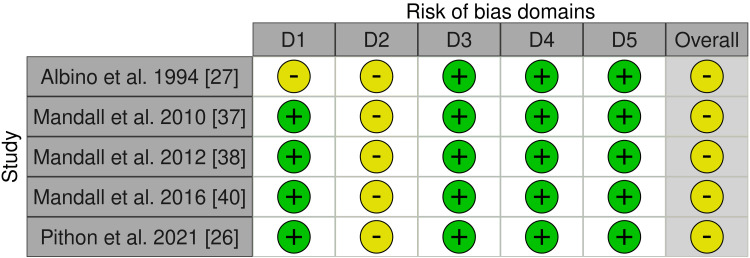
Risk of bias graph: The review authors’ judgments about each item's risk of bias for the included RCTs Domains: D1: Bias arising from the randomization process D2: Bias due to deviations from the intended intervention D3: Bias due to missing outcome data D4: Bias in the measurement of the outcome D5: Bias in the selection of the reported result Judgment: Yellow circle: Some concerns Green circle: Low risk of bias

**Figure 3:**
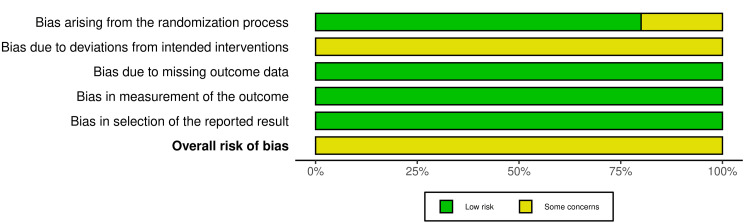
Risk of bias summary: The review authors’ judgments about each item's risk of bias, presented as percentages across all the included RCTs

Figures 2-3 display an overview of the included RCTs' overall risk of bias. The five included RCTs were classified as having some concern of bias [26,27,37,38,40]. Participants’ blinding was the most problematic field for all these trials. Moreover, the random sequence generation was unclear in Albino et al.'s study, reflecting some concern of bias in the randomization process [27]. More details about the risk of bias assessment of the included RCTs are given in Appendix 4.

**Figure 4:**
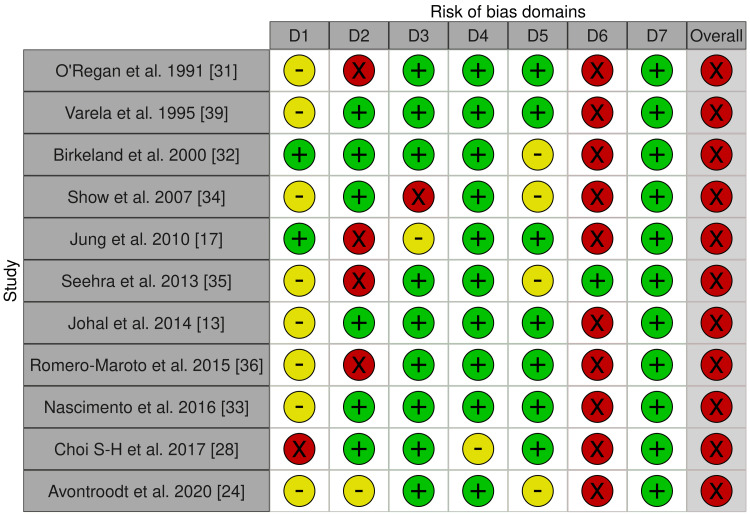
Risk of bias graph: The review authors’ judgments about each item's risk of bias for the included non-RCTs Domains: D1: Bias due to confounding D2: Bias due to selection of participants D3: Bias in the classification of interventions D4: Bias due to the deviations from intended interventions D5: Bias due to missing data D6: Bias in the measurement of outcomes D7: Bias in the selection of the reported result Judgment: Red circle: Serious risk of bias; Yellow circle: Some concerns; Green circle: Low risk of bias

**Figure 5:**
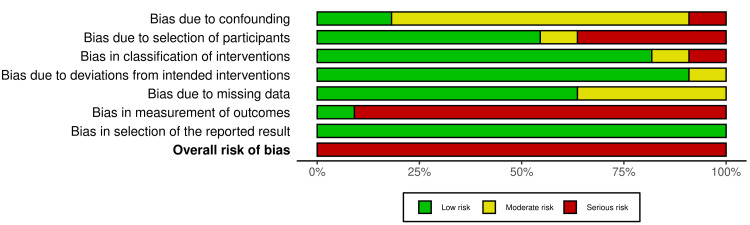
Risk of bias summary: The review authors’ judgments about each item's risk of bias are presented as percentages across all the included non-RCTs.

For non-RCTs, all of them were at serious risk of bias [13,17,24,28,31-36,39]. Bias in the measurement of outcomes was the most problematic field in most of the studies [13,17,24,28,31-34,36,39], due to the outcome assessors being aware of the intervention received by study participants. Figures 4-5 summarize the overall risk of bias in the non-RCT-included studies. More details about the risk of bias assessment are given in Appendix 5.

Effects of Interventions: Effect of Orthodontic Treatment on Self-Esteem

Sixteen studies assessed the influence of orthodontic treatment on patient SE in this systematic review. Only six studies have reported a significant increase in patient SE scores after orthodontic treatment (P<0.05) in these studies [13,17,26,32-34]. On the other hand, no statistically significant difference in SE scores following orthodontic treatment was observed in the other 10 studies [24,27,28,31,35-40]. Low-quality evidence supported this outcome based on the GRADE approach (Table 2).

**Table 2:** Summary of the findings table according to the GRADE guidelines for the included trials CI: confidence interval, PGD: parallel-group design, CSS: cross-sectional design a. Decline one level for risk of bias (some concern risk of bias [26,27,37,38,40] and high risk of bias [13,17,24,28,31-36,39]), one for inconsistency*, one for indirectness*** b. Decline one level for risk of bias (some concern risk of bias [26,27] and high risk of bias [13,17,24,28,31-33,35,36,39]), one for inconsistency*, one for indirectness***, and one for imprecision ** c. Decline one level for risk of bias (some concern risk of bias [37,38,40]) d. Decline one level for risk of bias (high risk of bias [17,32]), one for inconsistency*, one for indirectness***, and one for imprecision ** e. Decline one level for risk of bias (high risk of bias [17,24,31,32,36]), one for inconsistency*, one for indirectness***, and one for imprecision ** f. Decline one level for risk of bias (high risk of bias [24,36]), one for inconsistency*, one for indirectness***, and one for imprecision ** * Wide variance of point estimates across studies ** Limited number of trials *** Interventions delivered differently in different settings

Quality assessment criteria						Summary of the findings				Comments
No. of studies	Risk of bias	Inconsistency	Indirectness	Imprecision	Other considerations	No. of patients	Effects		Certainty	
							Absolute (95% CI)	Relative (95% CI)		
Effect of orthodontic treatment on self-esteem										
5 RCT (PGD) 7 cohort studies 4 CSS	Serious	Serious	serious	Not Serious	None	6287	-	-	⨁◯◯◯ ^a^ low	Six studies showed a significant increase in self-esteem after treatment (p
The effect of type of orthodontic treatment on self-esteem: Fixed orthodontic appliances:										
2 RCT (PGD) 6 cohort studies 4 CSS	Serious	serious	serious	Serious	Serious	1122	-	-	⨁◯◯◯ ^b^ Very Low	Five studies showed a significant increase in self-esteem after treatment (p
Facemask and bonded maxillary acrylic expansion device:										
3 RCT (PGD)	Serious	Not Serious	Not Serious	Not Serious	None	73	-	-	⨁⨁⨁◯^c^ Moderate	There was not a significant difference in self-esteem between the control and the experimental group.
Fixed versus removable orthodontic appliances										
1 cohort study 1 CSS	Serious	serious	Serious	Serious	Serious	4868	-	-	⨁◯◯◯ ^d^ Very Low	Fixed orthodontic treatment had a more significant effect on self-esteem than the removable appliances treatment.
The effect of gender on self-esteem:										
3 cohort study 2 CSS	Serious	serious	Serious	Serious	Serious	5088	-	-	⨁◯◯◯ ^e^ Very Low	
The effect of age on self-esteem:										
2 cohort study	Serious	serious	Serious	Serious	Serious	496	-	-	⨁◯◯◯ ^f^ Very Low	

First: The Effect of Type of Orthodontic Treatment on Self-Esteem

The fixed orthodontic appliances: The effect of fixed orthodontic treatment on patient SE was studied by 12 studies with different types of malocclusion [13,17,24,26-28,31-33,35,36,39]. A significant increase in SE scores was reported in five studies [13,17,26,32,33]. However, in the other seven studies, no statistically significant differences were noted in patients’ SE scores [24,27,28,31,35,36,39]. The strength of the evidence supporting this outcome, according to the GRADE approach, was low.

Facemask and bonded maxillary acrylic expansion device: Treatment with face masks and bonded maxillary acrylic expansion devices was evaluated by Mandall et al. [37] in three trials and over a long period (six years of follow-up). Tiny changes in SE over time as a result of protraction facemask treatment have been reported, and no statistically significant increase in SE score of children patients with class III malocclusion was found after 15 months, three years, and six years of treatment compared to baseline (P=0.22, P=0.56, P=0.48, respectively) [38,40]. The strength of the evidence supporting this outcome was moderate, based on the GRADE approach.

The fixed versus removable orthodontic appliances: Jung [17] and Birkeland et al.'s [32] studies investigated the impact of fixed and removable orthodontic treatment on SE in adolescent patients aged 11-16. They found that fixed orthodontic treatment had a more significant effect on SE (P=0.009, P<0.05, respectively) compared to the removable appliances treatment, as no significant increase in SE score was observed after treatment with these appliances (P=0.75, P>0.05, respectively). Notably, no information was reported in these studies about the types of malocclusions, types of removable appliances used, or duration of treatment. The strength of the evidence supporting this outcome was low, based on the GRADE approach.

Second: The effect of age and gender on SE: The relationship between patients’ sex and SE after orthodontic treatment was evaluated in five studies [17,24,31,32,36]. Two assessed the effect of both patients’ ages and gender on SE [24,36]. In regards to patients’ gender, Jung [17] noted that SE index (SI) increased in girls after fixed appliances treatment (SI=2.71±0.45, 2.86±0.43 in the untreated group, and after the fixed orthodontic treatment group, respectively, P<0.05). However, for the boys, orthodontic treatment did not affect SE levels (SI=2.80±0.47, 2.89±0.48 in the untreated group, and after fixed orthodontic treatment group, respectively, P>0.05) [17]. In contrast, Birkeland et al. [32] found that more girls than boys had developed negative self-evaluation after orthodontic treatment (P<0.001). Avontroodt et al.'s study on adolescents showed a decrease in SE levels for females and an increase for males between baseline and after 12 months of treatment [24]. The same results were also reported by O’Regan et al.'s study, as girls had lower SE than boys after orthodontic treatment [31]. Despite that, different results were reported by Romero-Maroto et al. in adult patients where no correlation between SE and gender was found [36]. Very low-quality evidence supported this outcome based on the GRADE approach. Regarding patients’ age, the Avontroodt et al. study showed that younger children had an improvement or stabilization in self-perception, whereas a decreased self-perception was found for older children [24]. On the other hand, according to Romero-Maroto et al., age did not have a significant correlation with SE, and it did not appear to be a relevant variable to consider [36]. Based on the GRADE approach, very low-quality evidence supported this outcome.

Discussion

Sixteen studies were included in this systematic review [13,17,24,26-28,31-40], assessing orthodontic treatment's impact on SE among many children, adolescents, and adult patients. Unfortunately, the interventions, the participants, the employed measurement scale, and the types of malocclusions were widely varied across these studies. Therefore, the results could not be pooled into a meta-analysis.

None of the included trials were judged to be at low risk of bias, and most were at high risk. This has affected the confidence in these findings, and the level or strength of evidence that can be gleaned from the included papers was relatively low.

Effect of Orthodontic Treatment on Self-Esteem

No agreement between the included studies was observed regarding the influence of orthodontic treatment on SE. Only six of the 16 included studies in this review have reported a significant increase in patients’ SE scores after orthodontic treatment procedures (P<0.05) [13,17,26,32-34]. This may be due to the higher satisfaction with dental appearance after fixed orthodontic treatment in these studies, which may positively affect SE. However, the other 10 trials have not observed any statistically significant difference in SE scores due to treatment [24,27,28,31,35-40]. This disagreement may be attributed to the fact that SE is a very complex topic that can change greatly during life’s stages. Moreover, it is not just impacted by one factor, such as malocclusion. Thus, there may be a range of interactions with orthodontic therapy.

The Effect of the Type of Orthodontic Treatment on Self-Esteem

The fixed orthodontic appliances: Twelve studies assessed the changes in SE levels due to treatment with fixed orthodontic appliances. There was uncertainty in the evidence as to whether or not there was an improvement in SE at the end of the treatment. A statistically significant increase in SE scores was reported due to treatment in five studies [13,17,26,32,33]. In contrast, in the other studies, no differences were reported [24,27,28,31,35,36,39]. This inconsistency may be attributed to the differences in the ages, demographic characteristics, types of malocclusions of the samples, and the absence of controlling for other confounder factors that could be responsible for part of the discrepancy between these studies.

Quick correct of teeth alignment can usually be achieved with fixed orthodontic treatment [17]; this may have a positive effect on a patient's SE, as the beautiful and well-aligned smile may boost patients’ confidence and improve their appearance, which can, in turn, improve their SE [41]. On the other hand, the effect of malocclusion on SE differs between people, depending on the personal perspective of the individual and his satisfaction with dental appearance, as some people consider dental appearance an important factor in their self-evaluation, while others see that dental appearance is not important and does not affect their self-evaluation [42].

Facemask and bonded maxillary acrylic expansion device: No significant increase in SE scores as a result of treatment with a face mask and a bonded maxillary acrylic expansion device in children with class III malocclusion was found by Mandall et al. over six years of follow-up [37,38,40]. This may be because of that the effect of orthopedic treatment alone was not strong enough to influence Piers-Harris scores, as it does not have an impact on teeth appearance [37]. It is also noteworthy that the questionnaire used in these studies does not include items specifically related to the face or teeth, and it is not designed to assess SE in these specific areas [20].

The fixed versus removable orthodontic appliances: As expected, removable orthodontic appliances had less effect on SE than fixed orthodontic appliances, according to Jung [17] and Birkeland et al. [32]. Usually, malocclusion cannot be completely corrected by removable appliance treatment [17]. Thus, psychological improvement might not be observed if some malocclusion still existed.

The Effect of Gender and Age on Self-Esteem

A few studies evaluated the effect of patients’ gender on SE after orthodontic treatment [17,24,31,32,36]. All of these studies were conducted on adolescent patients between 11 and 16 years of age, except for the study of Romero-Maroto et al., which included adult patients with a mean age of 29.80±9.55 years [36]. The results of this factor were different and somehow opposed between these studies. Therefore, the relationship between SE and patients’ gender cannot be emphasized in this review due to this disagreement. Both males and females who feel physically attractive tend to have higher SE [43]; however, many studies have shown that, during adolescence, girls’ attitudes about their appearance become more negative [44]. This difference between girls and boys may be because females are usually more conscious of their body image as the standards of aesthetics and beauty are more clearly defined for them [45]. This decline in girls’ perceived physical attractiveness is supposed to affect SE negatively [46]. This may also be reflected in orthodontic treatment, as females were reported to have greater concerns at the start and higher expectations at the end of treatment than males [47]. This may explain the results of Avontroodt et al., Birkeland et al., and O’Regan et al. studies, as females, had lower SE after orthodontic treatment than males [24,31,32].

There was insufficient evidence about the relationship between patients’ age and SE score after orthodontic treatment. Only two cohort trials evaluated this variable after fixed orthodontic treatment [24,36] and reported conflicting results. According to Avontroodt et al.'s study on adolescents, an inverse relationship may exist between patients’ age at the start of treatment and SE after treatment in studied subjects. This result disagrees with previous reviews about the development of SE over age in normal persons that have found an increase in SE from adolescence to middle adulthood [48]. Therefore, these results may suggest that early initiation of orthodontic treatment positively impacts SE more than in 14-year-old adolescents [24]. Romero-Maroto et al.'s study on adult patients reported that age had no significant correlation with SE. This difference with the previous study of Avontroodt et al. could be explained by the difference in the patients' ages (adolescents versus adults, respectively) between these studies. 

Limitations of the current review

One main limitation of this review is that only a small number of RCTs were included; all of them, including non-RCTs, were at moderate-to-serious risk of bias. This has affected the degree of confidence in the findings obtained. Another limitation of this systematic review was the variations between the included studies regarding the type of malocclusion, method of SE assessment, and assessment times. Hence, the results could not be pooled into a meta-analysis to provide an accurate estimate of the treatment effect. In addition, the effect of gender and age on patients' SE could not be confirmed across the included studies, and more studies are needed to establish good evidence in this field.

## Conclusions

There is low-quality evidence indicating that orthodontic treatment can improve patients’ self-esteem at the end of treatment. Results are conflicting about the effect of orthodontic treatment with fixed appliances on self-esteem. However, treatment with these appliances has a greater effect on self-esteem than that with removable appliances. Low-quality evidence supports these results. The influence of patients’ gender or age on self-esteem after orthodontic treatment is not clear. Further well-conducted studies using validated measurement scales of self-esteem are required to arrive at more robust conclusions with attention paid to the gender and age effect and the need for long-term follow-up periods.

## Appendices

**Table 3:** Appendix 1: Electronic search strategy used in the current review

Database	Search Strategy
CENTRAL (The Cochrane Library)	#1 malocclusion OR Class I OR Class II OR Class III OR overjet OR overbite OR crowding OR spaces OR protrusion OR retrognathism OR malalignment OR orthodontic* OR Orthodontic treatment OR Orthodontic therapy OR fixed appliances OR removable appliances OR myofunctional appliances OR children OR adolescence OR adults #2 Self OR Self-esteem OR SE OR self-perception OR Rosenberg’s self-esteem scale OR RSE OR Harter’s self-perception profile OR SPPC OR the Global Negative Self-evaluation OR the Self-esteem inventory OR SEI #3 #1 AND #2
EMBASE	#1 malocclusion OR Class I OR Class II OR Class III OR overjet OR overbite OR crowding OR spaces OR protrusion OR retrognathism OR malalignment OR orthodontic* OR Orthodontic treatment OR Orthodontic therapy OR fixed appliances OR removable appliances OR myofunctional appliances OR children OR adolescence OR adults #2 Self OR Self-esteem OR SE OR self-perception OR Rosenberg’s self-esteem scale OR RSE OR Harter’s self-perception profile OR SPPC OR the Global Negative Self-evaluation OR the Self-esteem inventory OR SEI #3 #1 AND #2
PubMed	#1 malocclusion OR Class I OR Class II OR Class III OR overjet OR overbite OR crowding OR spaces OR protrusion OR retrognathism OR malalignment OR orthodontic* OR Orthodontic treatment OR Orthodontic therapy OR fixed appliances OR removable appliances OR myofunctional appliances OR children OR adolescence OR adults #2 Self OR Self-esteem OR SE OR self-perception OR Rosenberg’s self-esteem scale OR RSE OR Harter’s self-perception profile OR SPPC OR the Global Negative Self-evaluation OR the Self-esteem inventory OR SEI #3 #1 AND #2
Google Scholar	#1 (malocclusion OR Class I OR Class II OR Class III OR overjet OR overbite OR crowding OR spaces OR protrusion OR retrognathism OR malalignment OR orthodontic* OR Orthodontic treatment OR Orthodontic therapy OR fixed appliances OR removable appliances OR myofunctional appliances OR children OR adolescence OR adults) AND (Self OR Self-esteem OR SE OR self-perception OR Rosenberg’s self-esteem scale OR RSE OR Harter’s self-perception profile OR SPPC OR the Global Negative Self-evaluation OR the Self-esteem inventory OR SEI)
Scopus	#1 TITLE-ABS-KEY (malocclusion OR Class I OR Class II OR Class III OR overjet OR overbite OR crowding OR spaces OR protrusion OR retrognathism OR malalignment OR orthodontic* OR Orthodontic treatment OR Orthodontic therapy OR fixed appliances OR removable appliances OR myofunctional appliances OR children OR adolescence OR adults). #2 TITLE-ABS-KEY (Self OR Self-esteem OR SE OR self-perception OR Rosenberg’s self-esteem scale OR RSE OR Harter’s self-perception profile OR SPPC OR the Global Negative Self-evaluation OR the Self-esteem inventory OR SEI) #3 #1 AND #2
Web of Science	#1TS= (malocclusion OR Class I OR Class II OR Class III OR overjet OR overbite OR crowding OR spaces OR protrusion OR retrognathism OR malalignment OR orthodontic* OR Orthodontic treatment OR Orthodontic therapy OR fixed appliances OR removable appliances OR myofunctional appliances OR children OR adolescence OR adults). #2TS= (Self OR Self-esteem OR SE OR self-perception OR Rosenberg’s self-esteem scale OR RSE OR Harter’s self-perception profile OR SPPC OR the Global Negative Self-evaluation OR the Self-esteem inventory OR SEI). #3TS= #1 AND #2
Trip	(malocclusion OR Class I OR Class II OR Class III OR overjet OR overbite OR crowding OR spaces OR protrusion OR retrognathism OR malalignment OR orthodontic* OR Orthodontic treatment OR Orthodontic therapy OR fixed appliances OR removable appliances OR myofunctional appliances OR children OR adolescence OR adults) AND (Self OR Self-esteem OR SE OR self-perception OR Rosenberg’s self-esteem scale OR RSE OR Harter’s self-perception profile OR SPPC OR the Global Negative Self-evaluation OR the Self-esteem inventory OR SEI)
OpenGrey	#1 orthodontic AND self-esteem #2 Self OR Self-esteem OR SE OR self-perception OR Rosenberg’s self-esteem scale OR RSE OR Harter’s self-perception profile OR SPPC OR the Global Negative Self-evaluation OR the Self-esteem inventory OR SEI

**Table 4:** Appendix 2: Keywords used in the search

Orthodontic	Malocclusion	Self-esteem
Orthodontic treatment	Class I	self-perception
Orthodontic therapy	Class II	self
Fixed appliances	Class III	Rosenberg’s self-esteem scale
Removable appliances	overjet	Harter’s self-perception profile
Myofunctional appliances	overbite	the Global Negative Self-evaluation
	Crowding	Self-esteem inventory
	Spaces	Piers Harris questionnaire
	malalignment	
	Protrusion	
	Retrognathism	

**Table 5:** Appendix 3: Excluded articles with reasons

Authors	Title	Reasons
Vulugundam et al. 2021	Is orthodontic treatment associated with changes in self-esteem during adolescence? A longitudinal study	Retrospective study
Majid et al. 2021	A comparison of self-esteem between patients undergoing fixed orthodontic treatment to those not receiving orthodontic treatment	This study did not meet the inclusion criteria for comparison: Group A: patients currently receiving no orthodontic treatment or the start of the treatment was less than six months. Group B: patients receiving orthodontic treatment in the past six months or more
Arrow et al. 2011	Quality of life and psychosocial outcomes after fixed orthodontic treatment: a 17-year observational cohort study	Retrospective study
Gazit-Rappaport et al. 2010	Psychosocial reward of orthodontic treatment in adult patients	Self-esteem was not evaluated
Vaida et al. 2009	Correlations between the changes in patients’ dentofacial morphology at the end of the orthodontic treatment and the psychological variables	Self-esteem was not assessed pre-treatment, and there is no comparison with a control group
Kenealy et al. 2007	The Cardiff dental study: A 20-year critical evaluation of the psychological health gain from orthodontic treatment	This study was the same as another article included in the review (A 20-year cohort study of health gain from orthodontic treatment: Psychological outcome), the same research team

**Table 6:** Appendix 4: Risk of bias assessment for randomized controlled trials according to the RoB-2 tool

Study	Randomization process	Deviations from intended interventions	Missing outcome data	Measurement of the outcome	Selection of the reported result	Over-all bias
Albino et al. 1994 [27]	Some concerns: No mention of the method used for randomisation “patients were randomly assigned to one of the study groups”(Page 84).	Some concerns: Blinding of participants and people delivering the intervention cannot be performed.	Low risk: No dropouts were reported.	Low risk: No details of blinding of outcome assessors. But we judge that the outcome was not likely to be influenced by knowledge of the intervention received	Low risk: The protocol was not registered. But the pre-defined outcomes mentioned in the methods section seemed to have been reported.	Some concerns
Mandall et al. 2010 [37]	Low risk: The randomization list was generated in randomization blocks of 10 with stratification according to gender. The computer-generated randomization sequence was concealed centrally	Some concerns: Blinding of participants and people delivering the intervention cannot be performed.	Low risk: 4 participants were excluded. missing outcome data occurred for reasons that are unrelated to the outcome	Low risk: The investigators performing the measurements and data analysis were blinded from the group assignments	Low risk: The protocol was not registered. However, the pre-defined outcomes mentioned in the methods section seemed reported.	Some concerns
Mandall et al. 2012 [38]	Low risk: The randomization list was generated in randomization blocks of 10 with stratification according to gender. The computer-generated randomization sequence was concealed centrally	Some concerns: Blinding of participants and people delivering the intervention cannot be performed.	Low risk: 10 participants were excluded. the result was not biased by missing outcome data.	Low risk: The investigators performing the measurements and data analysis were blinded from the group assignments”	Low risk: The protocol was not registered. However, the pre-defined outcomes mentioned in the methods section seemed reported.	Some concerns
Mandall et al. 2016 [40]	Low risk: The randomization list was generated in randomization blocks of 10 with stratification according to gender. The computer-generated randomization sequence was concealed centrally	Some concerns: Blinding of participants and people delivering the intervention cannot be performed.	Low risk: 8 participants were excluded. the result was not biased by missing outcome data	Low risk: The investigators performing the measurements and data analysis were blinded from the group assignments”	Low risk: The protocol was not registered. However, the pre-defined outcomes mentioned in the methods section seemed reported.	Some concerns
Pithon et al. 2021 [26]	Low risk: Randomization was performed by a researcher who was not involved in the clinical part of the study, using BioEstat 5.0 software	Some concerns: Blinding of participants and people delivering the intervention cannot be performed.	Low risk: No dropouts were reported.	Low risk: The investigators performing the measurements and data analysis were blinded from the group assignments”	Low risk: The protocol was not registered. However, the pre-defined outcomes mentioned in the methods section seemed reported.	Some concerns

**Table 7:** Appendix 5: Risk of bias for non-randomized trials according to the ROBINS-I tool

Study	Bias due to confounding	Bias in the selection of participants for the study	Bias in the classification of interventions	Bias due to deviations from intended interventions	Bias due to missing data	Bias in the measurement of outcomes	Bias in the selection of the reported result	Overall
O'Regan et al. 1991 [31]	Moderate: The post-treatment group was significantly older than the other groups.	Serious: Selection into the study was related (but not very strongly) to intervention and outcome	Low: Intervention status is well-defined	Low: No deviations from intended interventions were detected	Low: No dropouts were reported	Serious: No information about outcome assessors blinding and the results may be influenced by knowledge of the intervention received by study participants	Low: Pre-defined outcomes mentioned in the methods section seemed to have been reported	Serious
Varela et al. 1995 [39]	Moderate: Gender (female: male ratio), and a wide range of participants’ ages	Low: All participants who have been eligible for the target trial were included in the study	Low: Intervention status is well-defined	Low: No deviations from intended interventions were detected	Low: No dropouts were reported	Serious: No information about outcome assessors blinding and the results may be influenced by knowledge of the intervention received by study participants	Low: Pre-defined outcomes mentioned in the methods section seemed to have been reported.	Serious
Birkeland et al. 2000 [32]	Low: No confounding factors detected	Low: All participants who have been eligible for the target trial were included in the study	Low: Intervention status is well-defined	Low: No deviations from intended interventions were detected	Moderate outcome data were not available for all participants. Missing data were not related to the intervention	Serious: No information about outcome assessors blinding and the results may be influenced by knowledge of the intervention received by study participants	Low: Pre-defined outcomes mentioned in the methods section seemed to have been reported	Serious
Show et al. 2007 [34]	Moderate: Sociodemographic characteristics	Low: All participants who have been eligible for the target trial were included in the study	Serious: Intervention status is not well-defined	Low: No deviations from intended interventions were detected	Moderate outcome data were not available for all participants. Missing data were not related to the intervention	Serious: No information about outcome assessors blinding and the results may be influenced by knowledge of the intervention received by study participants	Low: Pre-defined outcomes mentioned in the methods section seemed to have been reported	Serious
Jung et al. 2010 [17]	Low: No confounding factors detected	Serious: Selection into the study was related (but not very strongly) to intervention and outcome	Moderate: Intervention status is well-defined, and some aspects of the assignments of intervention status were determined retrospectively	Low: No deviations from intended interventions were detected	Low outcome data available for all participants.	Serious: No information about outcome assessors blinding and the results may be influenced by knowledge of the intervention received by study participants	Low: Pre-defined outcomes mentioned in the methods section seemed to have been reported	Serious
Seehra et al. 2013 [35]	Moderate: Different types of malocclusions and small sample	Serious: the participants in this study were identified as bullied in the previous study	Low: Intervention status is well-defined	Low: No deviations from intended interventions were detected	Moderate outcome data were not available for all participants. Missing data were not related to the intervention	Low: No bias in the measurement of outcomes was detected	Low: Pre-defined outcomes mentioned in the methods section seemed to have been reported	Serious
Johal et al. 2014 [13]	Moderate: Gender (female: male ratio), and a wide range of participants’ ages	Low: All participants who have been eligible for the target trial were included in the study	Low: Intervention status is well-defined	Low: No deviations from intended interventions were detected	Low: No dropouts were reported	Serious: No information about outcome assessors blinding and the results may be influenced by knowledge of the intervention received by study participants	Low: Pre-defined outcomes mentioned in the methods section seemed to have been reported	Serious
Romero-Maroto et al. 2015 [36]	Moderate: Confounding factors may detected	Serious: Selection into the study was related (but not very strongly) to intervention and outcome	Low: Intervention status is well-defined	Low: No deviations from intended interventions were detected	Low No dropouts were reported	Serious: No information about outcome assessors blinding and the results may be influenced by knowledge of the intervention received by study participants	Low: Pre-defined outcomes mentioned in the methods section seemed to have been reported	Serious
Nascimento et al. 2016 [33]	Moderate: A wide range of participants’ ages	Low: All participants who have been eligible for the target trial were included in the study	Low: Intervention status is well-defined	Low: No deviations from intended interventions were detected	Low No dropouts were reported	Serious: No information about outcome assessors blinding and the results may be influenced by knowledge of the intervention received by study participants	Low: Pre-defined outcomes mentioned in the methods section seemed to have been reported	Serious
Choi S‐H et al. 2017 [28]	Serious: Different types of malocclusions with or without extraction, and a wide range of participants’ ages	Low: All participants who have been eligible for the target trial were included in the study	Low: Intervention status is well-defined	Moderate	Low: No dropouts were reported	Serious: No information about outcome assessors blinding and the results may be influenced by knowledge of the intervention received by study participants	Low: Pre-defined outcomes mentioned in the methods section seemed to have been reported	Serious
Avontroodt et al. 2020 [24]	Moderate: Sociodemographic characteristics	Moderate: The sample of adolescents was taken exclusively from the University Hospitals Leuven	Low: Intervention status is well-defined	Low: No deviations from intended interventions were detected	Moderate: Proportions of and reasons for missing participants were similar across intervention groups. The analysis addressed missing data and is likely to have removed any risk of bias.	Serious: No information about outcome assessors blinding and the results may be influenced by knowledge of the intervention received by study participants	Low: Pre-defined outcomes mentioned in the methods section seemed to have been reported	Serious
